# Handbook of the Diagnosis and Treatment of Skin Diseases

**Published:** 1884-12

**Authors:** 


**Handbook of the Diagnosis and Treatment of Skin Diseases.** By ARTHUR VAN HARLINGEN, M. D., Professor of Diseases of the Skin in the Philadelphia Polyclinic and Graduates in Medicine. With two colored plates. Philadelphia: P. Blakiston, Son & Co., 1012 Walnut St. 1884.

We are glad to welcome his little book of Dr. Van Harlingen. As a clinical teacher, those who have enjoyed his instructions know how thorough and painstaking he is. The book is, in its arrangement and practical bearing, peculiarly fitted for the general practitioner, and a careful perusal of it would save many physicians from maltreating cases of skin diseases which present themselves to them.

